# Hyperinvasiveness of *Listeria monocytogenes* sequence type 1 is independent of lineage I‐specific genes encoding internalin‐like proteins

**DOI:** 10.1002/mbo3.790

**Published:** 2019-01-17

**Authors:** Bulent Gözel, Camille Monney, Lisandra Aguilar‐Bultet, Sebastian Rupp, Joachim Frey, Anna Oevermann

**Affiliations:** ^1^ Division of Neurological Sciences, Vetsuisse Faculty University of Bern Bern Switzerland; ^2^ Graduate School for Cellular and Biomedical Sciences University of Bern Bern Switzerland; ^3^ Vetsuisse Faculty University of Bern Bern Switzerland

**Keywords:** cellular infection, host‐pathogen interaction, lineage 1, *Listeria monocytogenes*, sequence type, virulence factors

## Abstract

Listeriosis is a severe disease caused by the opportunistic bacterial pathogen *Listeria monocytogenes* (*L. monocytogenes*). Previous studies indicate that of the four phylogenetical lineages known, lineage I strains are significantly more prevalent in clinical infections than in the environment. Among lineage 1, sequence type (ST1) belongs to the most frequent genotypes in clinical infections and behaves hyperinvasive in experimental in vitro infections compared to lineage II strains suggesting that yet uncharacterized virulence genes contribute to high virulence of certain lineage I strains. This study investigated the effect of four specific lineage I genes encoding surface proteins with internalin‐like structures on cellular infection. CNS derived cell lines (fetal bovine brain cells, human microglia cells) and non‐CNS derived cell lines (bovine macrophage cells, human adenocarcinoma cells) that represent the various target cells of *L. monocytogenes* were infected with the parental ST1 strain and deletion mutants of the four genes. Despite their association with lineage I, deletion of the four genes investigated did not dampen the hyperinvasiveness of the ST1 strain. Similarly, these genes did not contribute to the intracellular survival and intercellular spread of *L. monocytogenes* ST1, indicating that these genes may have other functions, either during the infection process or outside the host.

## 1 INTRODUCTION

*Listeria* (*L.*) *monocytogenes* is an opportunistic bacterial pathogen, which is well adapted to both extracellular dwelling in outdoor and food factory environments (Carpentier & Cerf, 2011; Vivant, Garmyn, & Piveteau, 2013) and intracellular survival when ingested by a host (Vazquez‐Boland et al., 2001). In the host, *L. monocytogenes* may pass the intestinal tract without causing disease or may cause self‐limiting gastroenteritis (Gahan & Hill, 2014). However, when host barriers are crossed by invasion of cells, *L. monocytogenes* causes listeriosis, a potentially life‐threatening infection associated with septicemia, abortions, and neurological disease (Disson & Lecuit, 2012; Oevermann, Zurbriggen, & Vandevelde, 2010; Siegman‐Igra et al., 2002). Despite its low incidence rate, listeriosis is considered as a major public health threat due to the high fatality rate (Maertens de Noordhout et al., 2014; Swaminathan & Gerner‐Smidt, 2007).

Its remarkable niche adaptability is due to the large set of genes that allows *L. monocytogenes* to resist stressful environmental conditions and to invade and survive within phagocytic and non‐phagocytic host cells (Chaturongakul, Raengpradub, Wiedmann, & Boor, 2008; Kazmierczak, Mithoe, Boor, & Wiedmann, 2003). Many essential genes for intracellular survival of *L. monocytogenes* have been identified in strain EGD‐e, a widely used reference strain of the species, belonging to lineage II (Chakraborty, Hain, & Domann, 2000; Portnoy, Chakraborty, Goebel, & Cossart, 1992; Schnupf & Portnoy, 2007; Vazquez‐Boland et al., 2001). Six of them are located on the Listeria pathogenicity island number 1 (LIPI‐1), one of which is the transcriptional activator *prfA *(Scortti, Monzo, Lacharme‐Lora, Lewis, & Vazquez‐Boland, 2007; Vazquez‐Boland et al., 2001). *PrfA* regulates the transcription of the other 5 essential virulence genes on LIPI‐1 including *hly*, *plcA, plcB, mpl* (vacuolar escape), and *actA* (invasion, intracellular movement, intercellular spread, and avoidance of autophagy) and additionally other important genes outside of LIPI‐1 including *inlA* and *inlB* (Alvarez & Agaisse, 2016; Kanki, Naruse, & Kawatsu, 2018; Kocks et al., 1992; Phelps et al., 2018; Suarez, Gonzalez‐Zorn, Vega, Chico‐Calero, & Vazquez‐Boland, 2001). The proteins encoded by the latter two genes initialize bacterial internalization into non‐phagocytic cells by interacting with the two host membrane proteins E‐cadherin and c‐Met, respectively (Bierne & Cossart, 2007).

Studies indicate that strain diversity in *L. monocytogenes* is relevant in the context of infection and environmental survival. Recent epidemiological studies have shown that the relative prevalence of *L. monocytogenes* strains differs between clinical infection and environment (Dreyer et al., 2016; Maury et al., 2016; Orsi, Bakker, & Wiedmann, 2011). Of the four phylogenetical lineages known, lineage I strains are significantly more prevalent in clinical infection of both humans and ruminants than in the environment. This is particularly true for strains from specific sequence types (ST, as determined by multilocus sequence typing (MLST)), namely ST1 and 4. These two ST additionally behave hypervirulent and hyperinvasive in experimental in vivo and in vitro infections (Dreyer et al., 2016; Guldimann et al., 2015; Maury et al., 2016).

Previous studies from our group have shown that a ST1 strain from lineage I isolated from bovine rhombencephalitis (JF5203) behaves hyperinvasive compared to lineage II strains including EGD‐e (Dreyer et al., 2016; Rupp, Bartschi, Frey, & Oevermann, 2017) suggesting that, besides to the well‐known virulence genes, yet uncharacterized virulence genes may contribute to cellular invasion and virulence of certain strains. Therefore, the aim of this study was to assess the impact of lineage I‐specific virulence candidate genes on the cellular infection process. Emphasis was put on four genes of lineage I that encode surface proteins with internalin‐like structure. To investigate their effect on cellular infection, CNS‐derived cell lines (fetal bovine brain cells, human microglia cells) and non‐CNS‐derived cell lines (bovine macrophage cells, human adenocarcinoma cells) that represent the various target cells of *L. monocytogenes* were infected with a ST1 parental strain and deletion mutants derived thereof*.*

## 2 METHODS

### 2.1 Selection of candidate genes

For the identification of genes specific to lineage I, comparative whole genome analysis of 121 lineage I and 104 lineage II genomes was performed (Aguilar‐Bultet et al., 2018). One hundred and sixty‐seven genes were found to be present in lineage I genomes but absent from lineage II genomes (Aguilar‐Bultet et al., 2018). Four genes (LMJF5203_00388, LMJF5203_01291, LMJF5203_02767 and LMJF5203_02537) of 167 genes were chosen for analysis based on their internalin‐like protein structure (Appendix 1). All candidate genes specify for potential proteins with a leucine‐rich repeat (LRR) binding motif involved in host receptor recognition and interaction, which is found in internalin proteins (Bierne & Cossart, 2007). Furthermore, LMJF5203_00388, LMJF5203_01291, LMJF5203_02767 possess a LPXTG sortase recognition motif. Additionally, LMJF5203_00388 and LMJF5203_02537 are found in most strains of clonal complexes (CCs) belonging to lineage I and III, whereas LMJF5203_02767 and LMJF5203_01291 are present in most lineage I CCs but not in lineage III. None of the four genes was found in lineage II strains.

### 2.2 Expression of *Listeria monocytogenes *candidate genes by reverse‐transcription PCR

Virulence gene mRNA expression was assessed in stationary phase bacteria, which were used for the gentamicin protection assay. Liquid broth cultures were grown overnight, and bacteria were collected by centrifugation at 3,220 *g *for 5 min. Total RNA was extracted using RiboPure™ Bacteria kit (Ambion, Life technologies). Reverse transcription was performed using GoScript Reverse‐Transcription System (Promega) based on manufacturer's instruction. sigB and gyrA were used as control genes for RNA expression. DNA contamination was excluded by PCR using RNA that was not reverse‐transcribed. mRNA expression was assessed using following primers d0388_inside_fw, d0388_inside_rv, d1291_inside_fw, d1291_inside_rv, d2537_inside_fw, d2537_inside_rv, d2767_inside_fw, d2767_inside_rv, sigB_fw, sigB_rv, gyrA_fw, and gyrA_rv (Table 1), with amplification parameters as follows: initial denaturation at 95°C for 2 min, followed by 30 cycles of denaturation at 95°C for 2 min, annealing at 55°C for 30 s, and elongation at 72°C for 30 s, with a final extension at 72°C for 5 min.

**Table 1:** Primers used for cloning of pMAD and pHoss1 deletion plasmids and PCR analysis

**Primer**	**Sequence (5' ‐> 3')**
d00388_1_fw_SalI	TATATAGTCGACAGCATTACAGCAGCAGAAAACATC
d00388_2_rv	AAGTGTAAGCCCTTTGGATTTCATCTTGCTCC
d00388_3_fw	TCCAAAGGGCTTACACTTAGAAGAAAATAAAGG
d00388_4_rv_XmalI	ATATATCCCGGGGTTCAATGGGCGTCACTTGC
d0388_inside_fw	ATATTAACGACGCGCAAGTTACTG
d0388_inside_rv	TATAACCCTCTTTGACTGGGGTTG
d01291_1_fw_SalI	ATATATGTCGACGCTATCACCTGAAACTGAGGC
d01291_2_rv	GTTATGAGATCTTTTCATGATTAGTCTCCTTAGATG
d01291_3_fw	ATGAAAAGATCTCATAACTGCTGATAACATTTCTTG
d01291_4_rv_XmaI	ATATATCCCGGGTGTTTCATCATTATCCAGCGCC
d1291_inside_fw	AACTTGGTCGTCTGAAAGAA
d1291_inside_rv	TAAATCATCCGTTGTTTGCG
d02537_1_fw_SalI	TATATAGTCGACAATCAAGTTTGAAGTGGATGTACC
d02537_2_rv	ATTAATGGTATCTCCTCCAATTTATAAAGGACG
d02537_3_fw	GGAGGAGATACCATTAATTAATGGAAAACTTG
d02537_4_rv_XmaI	TATATACCCGGGTTGTAAATCAAACAGCAAAAAGCG
d2537_inside_fw	TCTCTAGGGTTGGGTTATTTTACC
d2537_inside_rv	AAATCCATACTTACCAAACTGTCC
d02767_1_fw_SalI	ATATATGTCGACCGAAACGATGCACTCATAACG
d02767_2_rv	TTTAAAGACTTCTTTGTAACACAGAAAAGCCC
d02767_3_fw	CAAAGAAGTCTTTAAAAGAAGTTAAACCACTCC
d02767_4_rv_XmaI	ATATATCCCGGGCGAAAGATTTGTTTAACGCTTATGG
d2767_inside_fw	TGAATATACCGTTACTGCTATCGG
d2767_inside_rv	TTTCTATAGGTAGGATGTGGTTGC
sigB_fw	GCGACGTTTGGGAAAAGCTT
sigB_rv	CGATGAAATCAGCAATGTCGCT
gyrA_fw	CGGTAAGTATCACCCCCACG
gyrA_rv	CGCGCTGGTAAAATGACTGG

Note

Restrictions sites are underlined.

### 2.3 Bacterial strains

*Listeria monocytogenes* strain JF5203 (NCBI Reference Sequence: NZ_LT985474.1; https://www.ncbi.nlm.nih.gov/nuccore/NZ_LT985474.1) belonging to phylogenetic lineage I, clonal complex 1, sequence type 1, isolated from a rhombencephalitis case in cattle was used as parental strain for cell invasion experiments and generation of the deletion mutants. *Listeria monocytogenes *strain EGD‐e, belonging to lineage II, clonal complex 9, sequence type 35 was used as a reference strain in order to confirm hyperinvasiveness of our parental strain. The deletion mutants LMJF5203_Δ00388 and LMJF5203_Δ02767 were generated using the pHoss1 plasmid, and LMJF5203_Δ01291 and LMJF5203_Δ02537 using the pMAD plasmid as previously described (Abdelhamed, Lawrence, & Karsi, 2015; Arnaud, Chastanet, & Debarbouille, 2004; Rupp et al., 2017). The upstream and downstream flanking regions of the genes of interest were amplified with the Expand High Fidelity Plus PCR system (Roche Diagnostics, Rotkreuz, Switzerland) using the amplification primer pairs d00388_1_fw_SalI/d00388_2_rv; d00388_3_fw/d00388_4_rv_XmaI; d01291_1_fw_SalI/d01291_2_rv; d01291_3_fw/d01291_4_rv_XmaI; d2416_1_fw_SalI/d 02537_2_rv; d2416_3_fw/d 02537_4_rv_XmaI; d02767_1_fw_SalI/d02767_2_rv; and d02767_3_fw/d02767_4_rv_XmaI (Table 1). Subsequently, amplicons of the flanking regions were joined via overlap extension PCR with the primer pairs d00388_1_fw_SalI/d00388_4_rv_XmaI; d01291_1_fw_SalI/d01291_4_rv_XmaI; d02537_1_fw_SalI/d02537_4_rv_XmaI; and d02767_1_fw_SalI/d02767_4_rv_XmaI. The fused DNA fragments were inserted into the SalI‐ and XmaI‐digested pMAD and pHoss1 plasmids, respectively, by ligation with T4 ligase to create pMad_Δ01291, pMad_Δ02537, pHOSS_Δ00388, and pHOSS_Δ02767. The parental strain JF5203 was transformed with these deletion plasmids as described (Abdelhamed et al., 2015; Arnaud et al., 2004). Deletion of the genes of interest was confirmed by colony PCR using the flanking region primer pairs as described above and additional primers binding to the ORF regions of the deleted genes (Table 1).

### 2.4 Whole genome sequencing of deletion mutants

*Listeria monocytogenes *mutants were grown overnight at 37°C in Bacto Brain Heart Infusion (BHI, Chemie Brunschwig, 237500), and genomic DNA was extracted using the DNA extraction kit (Invitrogen, PureLink™ Microbiome, DNA purification Kit, A29789). The whole genomes of the mutant strains were sequenced in GATC Biotech on an Illumina® HiSeq 4000 (150 bp paired‐end reads) platform according to the manufacturer's protocols. Genome coverage was between 200x and 300x. The Illumina reads of the different mutants were mapped to the whole genome of the parental strain in the Geneious software (Geneious 8.1.9, Biomatters Limited) to check the targeted deletion and to exclude spontaneous off‐target mutations (NCBI Access number: PRJNA504399; https://www.ncbi.nlm.nih.gov/bioproject/PRJNA504399).

### 2.5 Mammalian cell lines

The bovine macrophage cell line (BoMac), the human microglia cell line (HMC‐3), and the human epithelial colorectal adenocarcinoma cell line (Caco‐2) were grown in Dulbecco's modified Eagle's medium (DMEM) with Glutamax (Life Technologies, Zug, Switzerland) supplemented with 10% fetal calf serum (FCS) (Bioswisstec, Schaffhausen, Switzerland), 100U/ml penicillin and 10 µg/ml streptomycin (Life Technologies). Fetal bovine brain cells (FBBC‐1) were grown in a DMEM/F12 mix (1:1, Life Technologies) supplemented with 10% FCS, 50 ng/ml epithelial growth factor, 50 ng/ml recombinant human basic fibroblast growth factor (bFGF) (Sigma‐Aldrich, Buchs, Switzerland), 100 U/ml penicillin, 10 µg/ml streptomycin, and 1x N2 supplement (Life Technologies).

### 2.6 Axenic growth in broth

Single colonies of mutant strains and the parental strain were inoculated into BHI‐broth and grown overnight. The following day, fresh broth was inoculated with overnight culture at an OD600 of 0.05, and the OD600 was measured every 30 min for 7 hr. Bacterial growth was quantified in three independent experiments. Growth curves were fitted using a logarithmic scale in base 10, and generation time was calculated.

### 2.7 Gentamicin protection assay

Cells were grown to confluency in 24‐well plates with DMEM medium supplemented with 10% FCS and without penicillin/streptomycin. FBBC‐1 cells were differentiated by incubation with 100 µM forskolin (Merck‐Millipore, Schaffhausen, Switzerland) during 18 hr prior to infection (Takenouchi, Iwamaru, Sato, Yokoyama, & Kitani, 2009). Cells were starved in DMEM medium without FCS during 1 hr before inoculation. Overnight cultures of bacteria were added at 10^6^ CFUs per well corresponding to a multiplicity of infection (m.o.i) of 5:1. One hour following inoculation, cells were washed twice with phosphate‐buffered saline (PBS) and DMEM medium supplemented with 10% FCS and 50 µg/ml gentamicin (Sigma‐Aldrich) was added. FBBC‐1 cells were further supplemented with 100 µM forskolin. At different time points (2, 4, 8, and 24 hr p.i.), cells were washed twice with PBS and then lysed with 0,5% ice‐cold Triton‐X100 (Sigma‐Aldrich) and finally plated on BHI‐plates in several dilutions (1:1, 1:10, 1:100, 1:1,000, 1:10,000) for CFU quantification. Resulting CFU numbers were normalized to the inoculum. At least three independent experiments using triplicates were performed. The 2‐hr time point was used as an indicator for cellular invasion (Gaillard, Berche, Mounier, Richard, & Sansonetti, 1987; Sabet, Lecuit, Cabanes, Cossart, & Bierne, 2005). To estimate the intracellular fitness of strains, the number of intracellular duplications and duplication time between time intervals was calculated according to d = t/3.3 log (n2/n1), with d = duplication time, t = time interval, n1 = intracellular cfu number at the beginning of the time interval, and n2 = intracellular cfu number at the end of the time interval.

### 2.8 Immunofluorescence

For microscopical assessment of the gentamicin protection assay, cells were grown on glass coverslips, which were coated with poly‐D‐lysine hydrobromide for Caco‐2 cells. Coverslips were removed from the 24‐wells plates at the time points indicated above and fixed in 4% paraformaldehyde (PFA Sigma‐Aldrich) for 30 min at room temperature (RT). The coverslips were then washed three times in PBS supplemented with 0.5% Tween (PBS‐T), and cells were permeabilized with 0.5% Triton X‐100 for 30 min at RT. In order to block nonspecific labeling, cells were incubated with PBS‐T containing 10% normal goat serum (Dako, Baar, Switzerland) for 30 min and then incubated with rabbit *Listeria *O antiserum (BD, Allschwil, Switzerland, 1:200) in PBS‐T with 10% of goat serum for 1 hr at RT. Coverslips were then washed three times in PBS‐T and then incubated with Alexa Fluor 488‐ conjugated goat anti‐rabbit IgG secondary antibody (Life technologies, 1:500) and with DAPI (Invitrogen, Carlsbad, CA, USA, T3604, 1:10,000) for one hour in the dark. Coverslips were washed three times with PBS‐T, rinsed with distilled water, dried and mounted on Superfrost Plus glass slides (Menzel‐Gläser) with Glycergel Mounting Medium (Dako, Glostrup, Denmark). Cell cultures were imaged using an Olympus Fluoview FV1000 confocal microscope (Olympus, Tokyo, Japan), equipped with 405‐nm and 488‐nm laser channels. The number of infection foci as a measure of invasion was quantified at 24 hr p.i. Additionally, size of 5 foci per strain was measured as an indicator of spread in the BoMac cell line. Three independent experiments were performed.

### 2.9 Statistical analyses

For comparison of intracellular CFU dynamics and axenic growth between deletion mutants and JF5203, nonparametric Kruskal–Wallis analyses followed by Dunn's multiple comparison and nonparametric Mann–Whitney tests were performed for each time point using GraphPad Prism (GraphPad Software, La Jolla California USA, www.graphpad.com).

## 3 RESULTS

**Figure 1:**
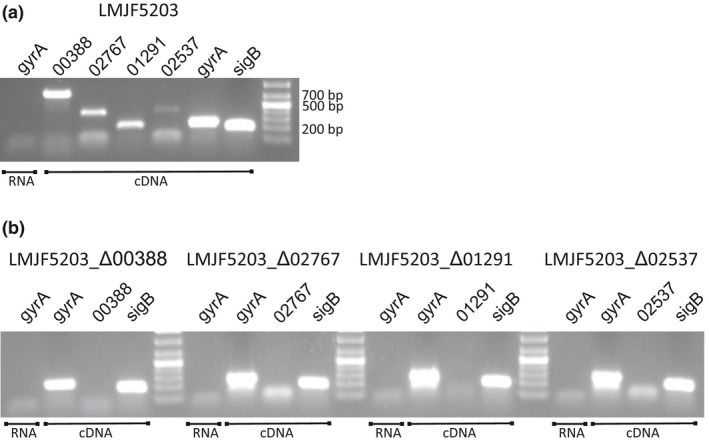
Expression of candidate genes in *Listeria monocytogenes* parental strain (LMJF5203) and deletion mutants. RNA was extracted from *L. monocytogenes* strains grown overnight in BHI‐broth, and expression of candidate and control genes (*gyrA* and *sigB*) was assessed by reverse‐transcription PCR. All genes are expressed in the parental strain (a). As expected, the candidate genes are not expressed in the respective deletion mutants (b)

This study aimed to investigate the putative involvement of lineage I‐specific genes encoding for internalin‐like proteins in the hyperinvasive behavior of lineage I strains. To this end, whole genome analysis of 121 lineage I and 104 lineage II genomes was realized and among 167 genes that were associated with lineage I, 4 internalin‐like genes were identified. A clinical ST1 (CC1, lineage I) strain, which is hyperinvasive compared to EGD‐e from CC9, lineage II (Appendices 2 and 3 and Rupp et al., 2017) expressed all four internalin‐like genes in vitro (Figure 1) and therefore was used to generate the respective deletion mutants (LMJF5203_Δ00388, LMJF5203_Δ01291, LMJF5203_Δ02537, and LMJF5203_Δ02767). Whole genome sequencing of the four deletion mutants and comparison to the parental strain confirmed the deletion and excluded the presence of spontaneous genomic off‐target mutations (results not shown). Reverse‐transcription PCR confirmed absence of expression in the deletion mutants (Figure 1). Mutants were tested for axenic growth in broth and in different cell lines representing different targets of *L. monocytogenes* (FBBC‐1 and HMC‐3 as model for CNS infection, BoMac and Caco‐2 as model for non‐CNS target cells).

### 3.1 Deletions do not affect fitness of *L. monocytogenes* mutants

**Figure 2:**
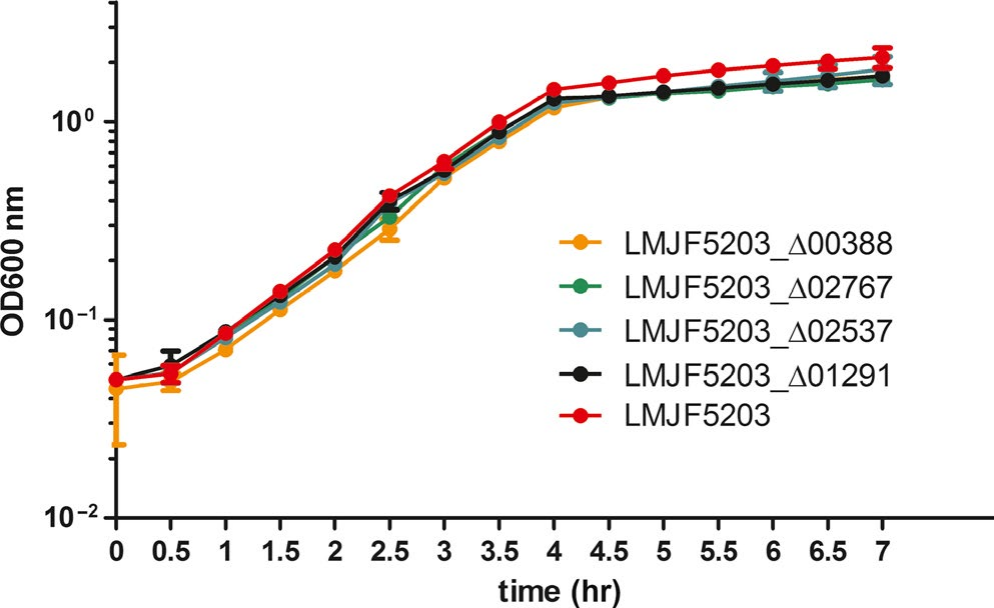
Axenic growth curve of the parental strain LMJF5203 and the four LMJF5203‐derived deletion mutants (LMJF5203_Δ00388, LMJF5203_Δ02767, LMJF5203_Δ02537, and LMJF5203_Δ01291). Strains were grown overnight in BHI medium at 37°C, inoculated into fresh broth at an OD600 of 0.05 and cultured at 37°C for 7 hr. The OD600 was measured every 30 min. Three independent experiments were performed. Results are expressed as mean, 95% CI. All mutants show a similar fitness as the parental strain

When grown in BHI medium, the four deletion mutants generated (LMJF5203_Δ00388, LMJF5203_Δ01291, LMJF5203_Δ02537, and LMJF5203_Δ02767) showed growth curves similar to the parental strain JF5203 indicating that the deletions did not exhibit any defect in extracellular growth and fitness (Figure 2).

### 3.2 Hyperinvasive behavior of ST1 is independent of LMJF5203_Δ00388, LMJF5203_Δ01291, LMJF5203_Δ02537, and LMJF5203_Δ02767

**Figure 3:**
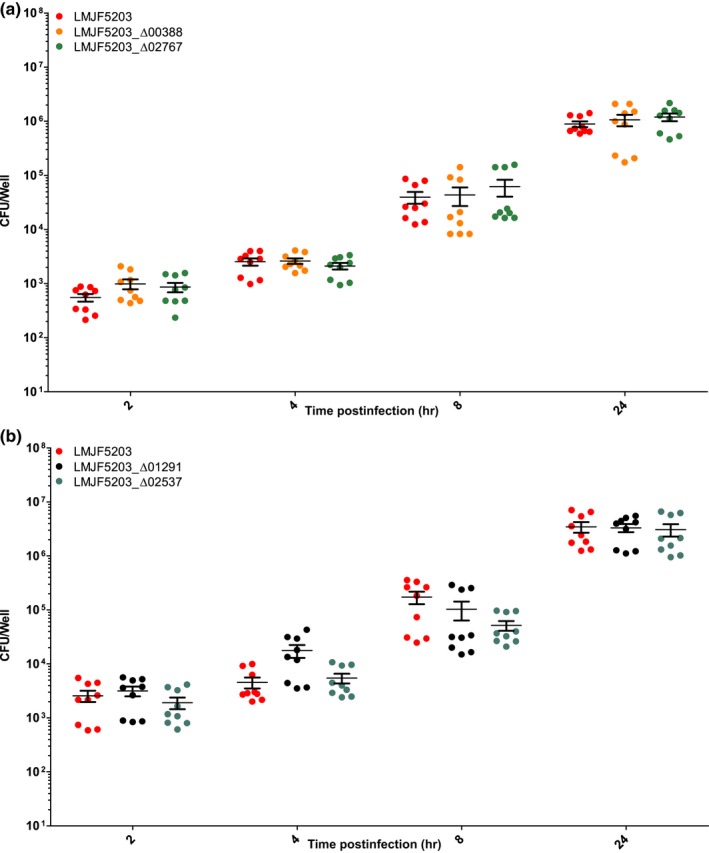
Infection of BoMac in the gentamicin exclusion assay in three independent experiments performed in triplicates. BoMac were infected with the indicated strains (LMJF5203_Δ00388, LMJF5203_Δ02767 (a), LMJF5203_Δ02537, and LMJF5203_Δ01291 (b)). At the indicated time points cells were lysed for CFU counting. Single CFU data are presented as dots, bars indicate the mean and error bars indicate the standard error of the mean (SEM). Statistical analysis (nonparametric Kruskal–Wallis test followed by Dunn's multiple comparison) did not reveal any significant difference between deletion mutants (LMJF5203_Δ00388, LMJF5203_Δ02767, LMJF5203_Δ02537, and LMJF5203_Δ01291) and parental strain

**Figure 4:**
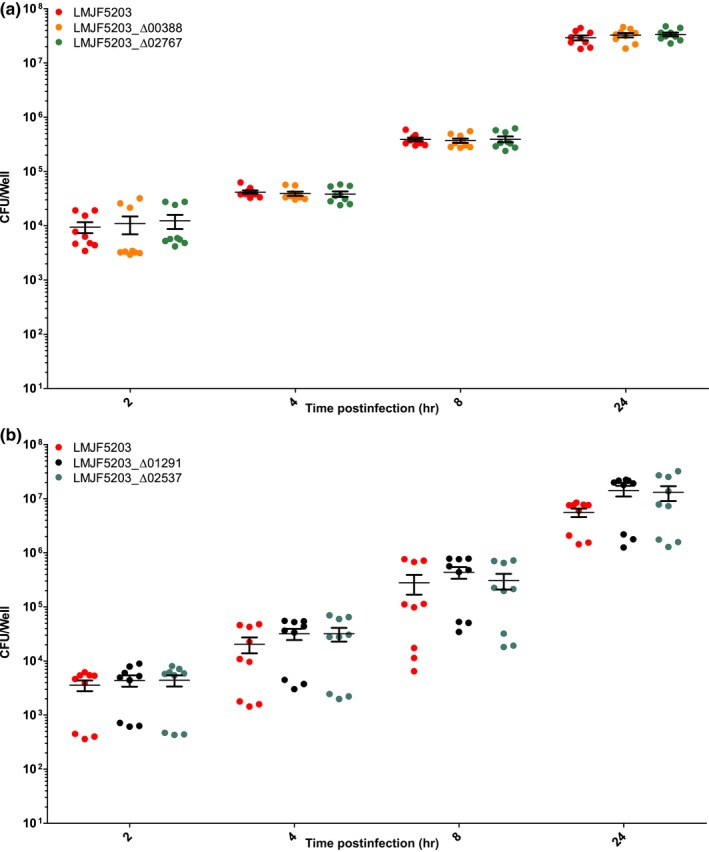
Infection of Caco‐2 in the gentamicin exclusion assay in three independent experiments performed in triplicates. Caco‐2 were infected with the indicated strains. At the indicated time points, cells were lysed for CFU counting. Single CFU data are presented as dots, bars indicate the mean, and error bars indicate the standard error of the mean (SEM). Statistical analysis (nonparametric Kruskal–Wallis test followed by Dunn's multiple comparison) did not reveal any significant difference between deletion mutants (LMJF5203_Δ00388, LMJF5203_Δ02767 (a), LMJF5203_Δ02537 and LMJF5203_Δ01291 (b)) and parental strain between 2 hr p.i and 24 hr p.i

**Figure 5:**
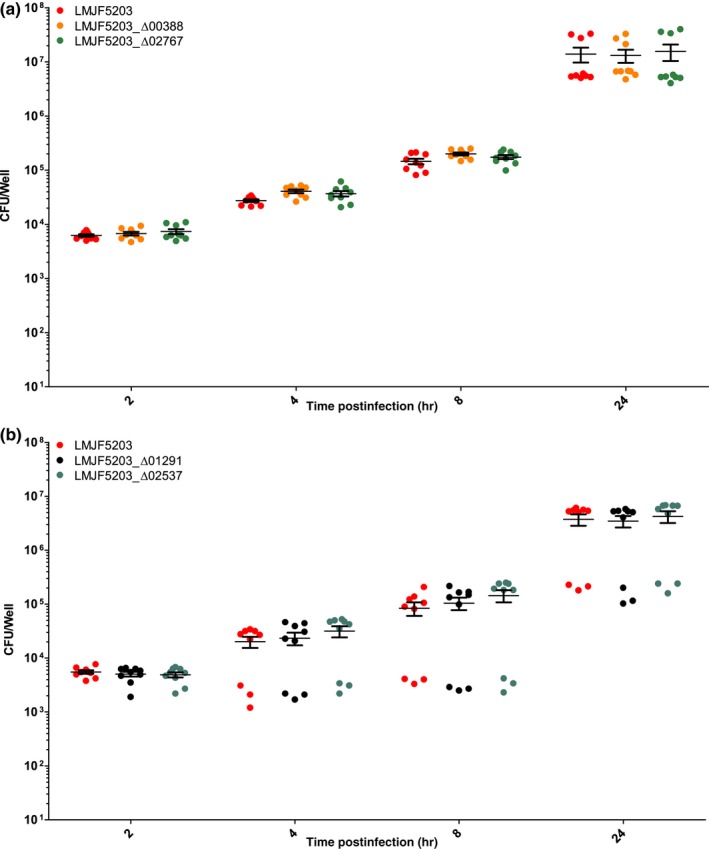
Infection of FBBC‐1 in the gentamicin exclusion assay in three independent experiments performed in triplicates. FBBC‐1 were infected with the indicated strains. At the indicated time points, cells were lysed for CFU counting. Single CFU data are presented as dots, bars indicate the mean, and error bars indicate the standard error of the mean (SEM). Statistical analysis (nonparametric Kruskal–Wallis test followed by Dunn's multiple comparison) did not reveal any significant difference between deletion mutants (LMJF5203_Δ00388, LMJF5203_Δ02767 (a), LMJF5203_Δ02537, and LMJF5203_Δ01291 (b)) and parental strain

**Figure 6:**
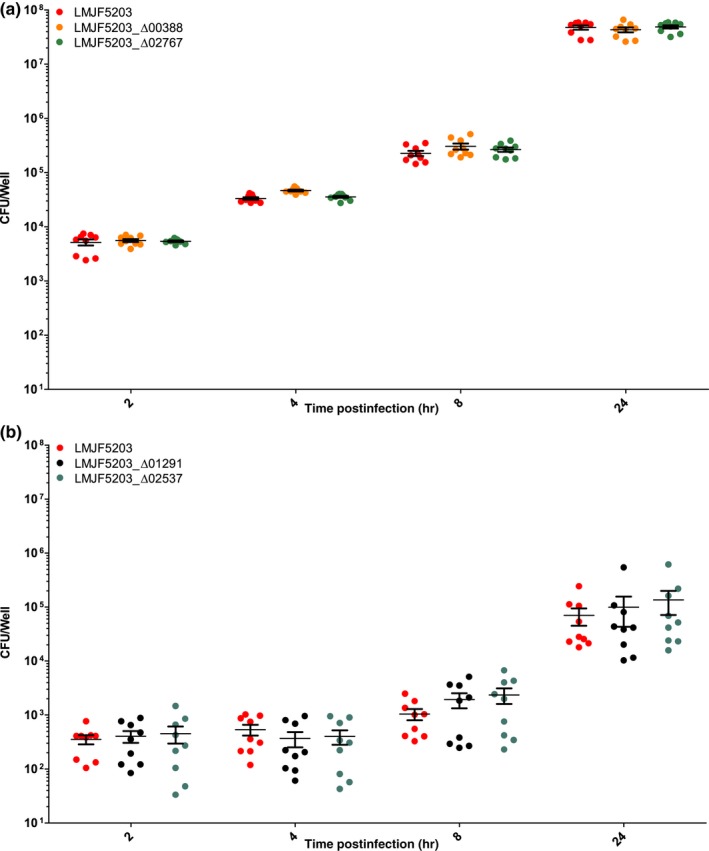
Infection of HMC‐3 in the gentamicin exclusion assay in three independent experiments performed in triplicates. HMC‐3 were infected with the indicated strains. At the indicated time points, cells were lysed for CFU counting. Single CFU data are presented as dots, bars indicate the mean and error bars indicate the standard error of the mean (SEM). Statistical analysis (nonparametric Kruskal–Wallis test followed by Dunn’s multiple comparison) did not reveal any significant difference between deletion mutants (LMJF5203_Δ00388, LMJF5203_Δ02767, LMJF5203_Δ02537, and LMJF5203_Δ01291) and parental strain

**Figure 7:**
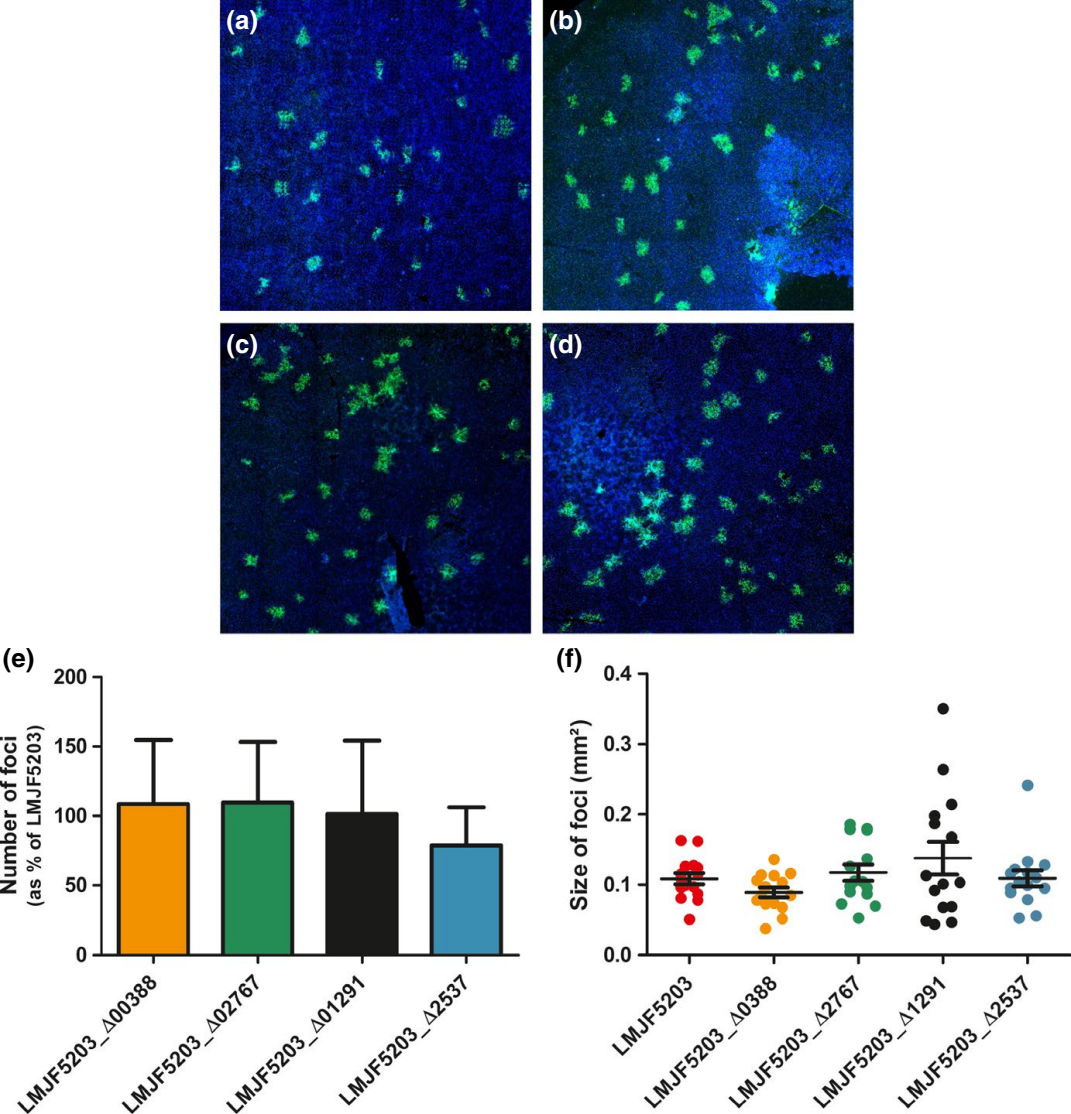
Immunofluorescence of BoMac 24 hr postinfection with different deletion mutants (a–d): (a) LM JF5203_Δ00388, (b) LMJF5203_Δ02537, (c) JF5203_Δ02767, (d) LMJF5203_Δ01291. *Listeria monocytogenes* are shown in green and nuclei in blue. There is no difference in number of foci per coverslip (e) and size of 5 foci per coverslip (f) at 24 hr p.i. in BoMac cells when compared to the parental strain JF5203

Confirming previous studies, the parental strain JF5203 (ST1, CC1) was hyperinvasive compared to EGD‐e (CC9) as indicated by higher CFU counts from 2 hr p.i. on and by the higher number of infection foci in the analyzed cover slips (Appendices 2, 3 and 3). The intercellular spread was similar between EGD‐e and JF5203 (Appendix 3). Deletion of LMJF5203_00388, LMJF5203_02767, LMJF5203_02537, and LMJF5203_01291 in the hyperinvasive strain JF5203 did not result in any significant reduction of invasion of various CNS and non‐CNS cell lines (Figures 3, 4, 5, 6) as indicated by similar CFU numbers at 2 hr p.i. Supporting these results, the number of infection foci as determined by immunofluorescence at 24 hr p.i. was similar between deletion mutants and parental strain (Figure 7a–e). Also, kinetics of intracellular duplication were similar to the parental strain as indicated by similar increase in CFU numbers at later timepoints (4, 8, and 24 hr, Appendix 4). None of the four deletions had an effect on the size and shape of infection foci in the BoMac cell line indicating that the genes are not involved in intercellular spread (Figure 7a–d,f).

## 4 DISCUSSION

*Listeria monocytogenes *has a clonal population structure that is organized in four phylogenetic lineages with lineages I and II being the major lineages (Maury et al., 2016; Nightingale, Windham, & Wiedmann, 2005). Within these two lineages, hyper‐ and hypovirulent clones have been identified (Orsi et al., 2011). Hypovirulent clones of *L. monocytogenes *generally belong to lineage II (Jacquet et al., 2004; Maury et al., 2016; McLauchlin, 1990; Orsi et al., 2011), while hypervirulent clones have been predominantly found in lineage I, which is also the most prevalent lineage in animal and human infection (Chenal‐Francisque et al., 2011; Jacquet et al., 2004; Kim et al., 2018; Maury et al., 2016; Orsi et al., 2011). Of those, strains belonging to sequence type (ST) 1 are particularly prevalent in CNS infection of ruminants and humans (Dreyer et al., 2016; Maury et al., 2016) and behave hyperinvasive in vitro compared to the reference strain EGD‐e and other strains from lineage II (Dreyer et al., 2016; Guldimann et al., 2015; Rupp et al., 2017). Differences in genomic gene content suggest that lineage I strains harbor specific genes that may confer hyperinvasion and hypervirulence resulting in the higher prevalence of these strains in clinical infection (Dreyer et al., 2016; Maury et al., 2016). Therefore, we investigated the impact of four genes encoding for surface proteins with internalin‐like structure (LMJF5203_00388, LMJF5203_02767, LMJF5203_02537 and LMJF5203_01291) (Aguilar‐Bultet et al., 2018) on cellular invasion, intracellular survival, and intercellular spread.

In this study, we confirmed that compared to strain EGD‐e our ST1 strain JF5203 is hyperinvasive in the different cell systems studied (Rupp et al., 2017) and that invasion is dependent on the infected cell line suggesting cell type‐specific interactions between *L. monocytogenes *and the host cell. Our results show that despite their association with lineage I, none of the four investigated genes is involved in the hyperinvasiveness of the ST1 strain, independently of the type and host origin of cell line infected (macrophages, microglia, fetal brain cells, colon adenocarcinoma epithelium). Additionally, these genes do not contribute to the intracellular survival and intercellular spread of *L. monocytogenes* ST1. Our results show that despite the LRR binding and LPXTG sortase recognition motifs, LMJF5203_00388, LMJF5203_02767, LMJF5203_02537, and LMJF5203_01291 do not act as invasins and may have other functions, either during the infection process or outside the host. Similar has been shown for the internalins InlC, InlH, and InlJ (Bierne & Cossart, 2007). Hence, other factors likely contribute to the hyperinvasiveness of ST1/CC1 and its high prevalence in clinical infections. Alternatively, the function of the investigated genes may be redundant with other genes of *L. monocytogenes* and therefore the phenotype not be picked up in our systems. Certainly, in vitro infection assays are limited tools for the study of virulence factors as they do not reflect all aspects of the infectious process in vivo. Indeed, other virulence factors (*llsB*, *inlJ*) were shown to be involved in in vivo infection, while no particular phenotype could be attributed to these virulence factors in in vitro infections of cell lines (Quereda, Andersson, Cossart, Johansson, & Pizarro‐Cerda, 2018; Quereda et al., 2017; Rupp et al., 2017; Sabet et al., 2008). Therefore, we cannot fully rule out an impact of these four genes on the infectious process either in other cell types, which were not represented in our study, or in more complex physiological systems including the immune system and host barriers where such membrane proteins may exhibit moonlighting functions on the bacterial cell surface (Copley, 2012). The cause for the association of these four internalin‐like genes with lineage I remains to be determined.

## CONFLICT OF INTEREST

The authors declare no conflict of interest.

## AUTHORS CONTRIBUTION

AO designed the study. BG, SR, and AO conceived and designed the experiments. LAB performed the whole genome comparisons. BG, SR, and CM performed the bacterial cloning and infection experiments. BG and AO wrote the manuscript. CM, SR, LAB, and JF critically revised the manuscript.

## ETHICS STATEMENT

None required.

## Data Availability

All data are included in the main manuscript. Raw data are available on request.

## Appendix 1

Amino acid sequences of LMJF5203_00388 (a), LMJF5203_01291 (b), LMJF5203_02537 (c), and LMJF5203_2767 (d). Domains are annotated in different colors (LPXTG Motif  Signal peptide  LRR (leucine rich repeat) ). Signal peptide and LPXTG were determined using https://www.expasy.org, LRR motifs using https://www.expasy.org for LMJF5203_00388 and LMJF5203_01291 and www.lrrfinder.com for LMJF5203_02537 and LMJF5203_2767.

## Appendix 2

Infection of BoMac (a), Caco‐2 (b), FBBC‐1 (c) and HMC‐3 (d) with LMJF5203 (red) and EGD‐e (blue) in the gentamicin exclusion assay. At the indicated time points, cells were lysed for CFU counting. Three independent experiments were performed in triplicates. Single CFU data are presented as dots, bars indicate the mean, and error bars indicate the standard error of the mean (SEM). Statistical analysis (nonparametric Mann–Whitney test) did reveal significant difference between EGD‐e and LMJF5203.

## Appendix 3

Immunofluorescence of BoMac after 24 hr of infection with (a) LMJF5203 and (b) EGD‐e. *Listeria monocytogenes* are shown in green and nuclei in blue. EGD‐e shows decreased foci numbers compared to the parental strain LMJF5203 (c) but similar foci size (d).

## Appendix 4

Fold change, number of duplications and duplication time of *Listeria monocytogenes* JF5203 and isogenic deletion mutants in BoMac, HMC‐3, FBBC‐1, and CaCo‐2.

**Table:**

Time interval				
Strain	2–4 hr	4–8 hr	8–24 hr	2–24 hr
BoMac				
Fold change				
LMJF5203	4,583582522	15,5596285	22,50513127	1605,039885
LMJF5203_Δ00388	2,651962832	16,66801311	24,31400553	1074,750801
LMJF5203_Δ02767	2,463174925	29,05865569	19,31753369	1382,682455
Number of duplications				
LMJF5203	2,196475648	3,95973571	4,492182075	10,64839343
LMJF5203_Δ00388	1,407060556	4,059010234	4,60371568	10,06978647
LMJF5203_Δ02767	1,300519086	4,860896057	4,271839009	10,43325415
Duplication time in minutes				
LMJF5203	54,63297538	60,61010571	213,7046059	123,9623619
LMJF5203_Δ00388	85,28417593	59,12771492	208,527213	131,0852026
LMJF5203_Δ02767	92,27084884	49,37361284	224,72757	126,5185321

**Table:**

Time interval				
Strain	2–4 hr	4–8 hr	8–24 hr	2–24 hr
BoMac				
Fold change				
LMJF5203	1,767764298	38,05098039	20,02135293	1346,739601
LMJF5203_Δ01291	5,585830102	5,842304537	32,27726472	1053,340148
LMJF5203_Δ02537	2,860383944	9,454748831	59,48329712	1608,678883
Number of duplications				
LMJF5203	0,821925928	5,24986172	4,323467562	10,39525521
LMJF5203_Δ01291	2,481771693	2,546537562	5,01244642	10,04075568
LMJF5203_Δ02537	1,51620881	3,241039133	5,894412712	10,65166066
Duplication time in minutes				
LMJF5203	145,9985576	45,71548981	222,0439928	126,9810094
LMJF5203_Δ01291	48,35255407	94,24561552	191,5232442	131,4642087
LMJF5203_Δ02537	79,1447716	74,05032465	162,8660983	123,9243384

**Table:**

Time interval				
Strain	2–4 hr	4–8 hr	8–24 hr	2–24 hr
HMC‐3				
Fold change				
LMJF5203	6,435560863	6,813925297	208,8039615	9156,352501
LMJF5203_Δ00388	8,348928598	6,519884194	141,51048	7702,988202
LMJF5203_Δ02767	6,58260637	7,449764417	183,3785193	8992,674766
Number of duplications				
LMJF5203	2,686065886	2,768486131	7,706005273	13,16055729
LMJF5203_Δ00388	3,061591071	2,70484634	7,14476509	12,9112025
LMJF5203_Δ02767	2,718658929	2,897194804	7,518680844	13,13453458
Duplication time in minutes				
LMJF5203	44,67500244	86,68997736	124,5781655	100,299704
LMJF5203_Δ00388	39,19530637	88,72962448	134,3641097	102,2367978
LMJF5203_Δ02767	44,13940958	82,83875136	127,6819724	100,4984221

**Table:**

Time interval				
Strain	2–4 hr	4–8 hr	8–24 hr	2–24 hr
HMC‐3				
Fold change				
LMJF5203	1,514319923	1,943310072	67,11636437	197,5095779
LMJF5203_Δ01291	0,907695924	5,250572548	51,50628647	245,4750107
LMJF5203_Δ02537	0,883355971	5,881794187	57,42170845	298,3470049
Number of duplications				
LMJF5203	0,598670028	0,958516114	6,068592663	7,625778806
LMJF5203_Δ01291	0,139719016	2,39247475	5,686676622	7,939432356
LMJF5203_Δ02537	0,178933169	2,556256303	5,84352435	8,220847484
Duplication time in minutes				
LMJF5203	200,4443088	250,3870269	158,1915369	173,0970742
LMJF5203_Δ01291	858,8666255	100,3145383	168,8156482	166,2587375
LMJF5203_Δ02537	670,6414487	93,88729907	164,2844185	160,5673871

**Table:**

Time interval				
Strain	2–4 hr	4–8 hr	8–24 hr	2–24 hr
FBBC‐1				
Fold change				
LMJF5203	4,39689568	5,320430045	95,81982574	2241,5492
LMJF5203_Δ00388	6,053174311	4,884853081	66,02136972	1952,177112
LMJF5203_Δ02767	5,006113685	4,730859638	89,36151736	2116,36858
Number of duplications				
LMJF5203	2,136485304	2,411542862	6,582252285	11,13028045
LMJF5203_Δ00388	2,597691896	2,288315172	6,044861165	10,93086823
LMJF5203_Δ02767	2,323691054	2,242102357	6,481581778	11,04737519
Duplication time in minutes				
LMJF5203	56,16701402	99,52134949	145,8467343	118,5953944
LMJF5203_Δ00388	46,19485481	104,8806576	158,8125804	120,7589344
LMJF5203_Δ02767	51,64197702	107,0423922	148,1119938	119,4853961

**Table:**

Time interval				
Strain	2–4 hr	4–8 hr	8–24 hr	2–24 hr
FBBC‐1				
Fold change				
LMJF5203	3,631826228	4,190975885	44,22098102	673,0829592
LMJF5203_Δ01291	4,627432239	4,470073756	33,17831577	686,2922478
LMJF5203_Δ02537	6,431270966	4,573448713	29,32636263	862,5788825
Number of duplications				
LMJF5203	1,860695175	2,06728622	5,466659126	9,394640521
LMJF5203_Δ01291	2,210211864	2,160298636	5,052168748	9,422679248
LMJF5203_Δ02537	2,685103875	2,193282473	4,874126239	9,752512587
Duplication time in minutes				
LMJF5203	64,49202512	116,0942291	175,6099983	140,5056422
LMJF5203_Δ01291	54,29343763	111,0957513	190,017406	140,0875447
LMJF5203_Δ02537	44,69100846	109,4250298	196,9583784	135,3497356

**Table:**

Time interval				
Strain	2–4 hr	4–8 hr	8–24 hr	2–24 hr
CaCo‐2				
Fold change				
LMJF5203	4,435299817	9,31248521	74,72648524	3086,477634
LMJF5203_Δ00388	3,572447031	9,506599778	87,59303322	2974,819191
LMJF5203_Δ02767	3,126713623	10,24715499	85,42528529	2737,019231
Number of duplications				
LMJF5203	2,149031631	3,219166229	6,223547762	11,59174562
LMJF5203_Δ00388	1,83691262	3,248929425	6,452744223	11,53858627
LMJF5203_Δ02767	1,644647088	3,357151512	6,416591256	11,41838986
Duplication time in minutes				
LMJF5203	55,83910365	74,55346599	154,2528533	113,8741345
LMJF5203_Δ00388	65,32700503	73,87048735	148,7739118	114,3987634
LMJF5203_Δ02767	72,96398167	71,48917739	149,6121479	115,6029893

**Table:**

Time interval				
Strain	2–4 hr	4–8 hr	8–24 hr	2–24 hr
CaCo‐2				
Fold change				
LMJF5203	5,738236211	13,60616922	19,96733907	1558,958242
LMJF5203_Δ01291	7,261276156	13,73673343	32,34485431	3226,27679
LMJF5203_Δ02537	7,228074597	9,665179038	42,3800878	2960,699849
Number of duplications				
LMJF5203	2,520607357	3,766189032	4,319570181	10,60636657
LMJF5203_Δ01291	2,860223122	3,77996707	5,015464309	11,6556545
LMJF5203_Δ02537	2,853611395	3,272796457	5,405314672	11,53172252
Duplication time in minutes				
LMJF5203	47,6075735	63,72489484	222,2443345	124,4535526
LMJF5203_Δ01291	41,95476888	63,49261662	191,4080015	113,2497536
LMJF5203_Δ02537	42,05197673	73,33178312	177,6029812	114,4668541
